# Candidalysin: Connecting the pore forming mechanism of this virulence factor to its immunostimulatory properties

**DOI:** 10.1016/j.jbc.2022.102829

**Published:** 2022-12-26

**Authors:** Charles M. Russell, Jennifer A. Rybak, Jian Miao, Brian M. Peters, Francisco N. Barrera

**Affiliations:** 1 Department of Biochemistry & Cellular and Molecular Biology, University of Tennessee, Knoxville, Tennessee, USA; 2 School of Genome Science and Technology, University of Tennessee, Knoxville, Tennessee, USA; 3 Graduate Program in Pharmaceutical Sciences, College of Graduate Health Sciences, University of Tennessee Health Science Center, Memphis, Tennessee, USA; 4 Department of Clinical Pharmacy and Translational Science, College of Pharmacy, University of Tennessee Health Science Center, Memphis, Tennessee, USA; 5 Department of Microbiology, Immunology, and Biochemistry, College of Medicine, University of Tennessee Health Science Center, Memphis, Tennessee, USA

**Keywords:** membrane pore, peptide, candidiasis, EGFR, Candida albicans, CL, candidalysin, ECE1, extent of cell elongation 1, EGFR, epidermal growth factor receptor, GI, gastrointestinal, MAPs, membrane active peptides, MAPK, mitogen-activated protein kinase, MMP, matrix metalloproteinase, OPC, oropharyngeal candidiasis, VVC, vulvovaginal candidiasis

## Abstract

*Candida albicans* is a deadly pathogen responsible for millions of mucosal and systemic infections per year. The pathobiology of *C. albicans* is largely dependent on the damaging and immunostimulatory properties of the peptide candidalysin (CL), a key virulence factor. When CL forms pores in the plasma membrane of epithelial cells, it activates a response network grounded in activation of the epidermal growth factor receptor. Prior reviews have characterized the resulting CL immune activation schemas but lacked insights into the molecular mechanism of CL membrane damage. We recently demonstrated that CL functions by undergoing a unique self-assembly process; CL forms polymers and loops in aqueous solution prior to inserting and forming pores in cell membranes. This mechanism, the first of its kind to be observed, informs new therapeutic avenues to treat *Candida* infections. Recently, variants of CL were identified in other *Candida* species, providing an opportunity to identify the residues that are key for CL to function. In this review, we connect the ability of CL to damage cell membranes to its immunostimulatory properties.

*Candida albicans* is typically a harmless, commensal fungus that can become a deadly pathogen. Under normal conditions, *C. albicans* exists as a benign resident of the human microbiota. However, shifts in normal environmental conditions can cause *C. albicans* to become pathogenic and harm its human host through infection of epithelial cells throughout the body (*i.e.*, oral, vaginal, and skin tissues) (1). Epithelial infection results in diseases such as oropharyngeal candidiasis (OPC or thrush) and vulvovaginal candidiasis (VVC), which cause inflammation, itchiness, and burning symptoms (2). Candidiasis disproportionately targets immunocompromised individuals and marginalized populations (1). Infections can also travel beyond surface epithelium, typically through the gut, and cause systemic infections, which have a striking mortality rate of 20 to 50% (1, 3, 4). Because of the major public health implications of *C. albicans*, it is important to understand the molecular mechanisms of pathogenicity. In this review, we discuss the peptide toxin candidalysin (CL), which has recently been shown to be essential in the ability of *C. albicans* to cause disease.

The transition to pathogenicity is initially characterized by a change in fungal morphology. Commensal *C. albicans* predominantly maintains an ovoid morphology, whereas the pathogenic form of the fungus grows filamentous hyphae that invade epithelial cells and allow for efficient escape from immune cell (*e.g.*, macrophage) phagocytosis (5, 6, 7). However, this morphological change is not sufficient to drive pathogenicity. A hypha creates an invasion pocket in which virulence factors are released to promote pathogenicity (8). It has become apparent that the most important of these virulence factors is CL. CL was identified through a genetic screen of virulence genes expressed during filamentation (4). The fungal gene extent of cell elongation 1 (*ECE1*) was identified to be required for epithelial cell damage and immune activation. Ece1 is a polypeptide that undergoes sequential rounds of proteolytic cleavage at lysine-arginine (KR) residues by the proteases Kex1/2 (9). These events result in the production of eight peptides. Moyes *et al.* (4) screened these peptides and identified Ece1_III_ (now called CL) to be the only one able to cause cell damage and immune activation.

Genetically altered strains of *C. albicans* devoid of CL can undergo the yeast-to-hypha switch but do not cause mucosal disease (4), emphasizing the crucial role of CL during infection. It has been observed that sustained activity of MAPK (mitogen-activated protein kinase) is the driving force of CL-dependent epithelial cell immune signaling (4, 10, 11, 12). MAPK signaling during *C. albicans* infection leads to increased levels of the transcription factor c-Fos, which causes the release of cytokines, including interleukin (IL)-1α, IL-1β, G-CSF, and GM-CSF (Fig. 1). These secreted inflammatory mediators function to recruit immune cells to the infection site. These cells can be beneficial (13) or detrimental (14) to the host, depending on the organ and disease state, as we will discuss below. Interestingly, despite the fact that CL is required for activation of immune signaling, so far, no cell surface binding partners for CL have been found that trigger signaling. Instead, it is believed that CL causes cell immune signaling solely through its ability to permeabilize the plasma membrane of the infected cell. Therefore, it is important understand the molecular mechanism of CL pore formation.

**Figure 1:**
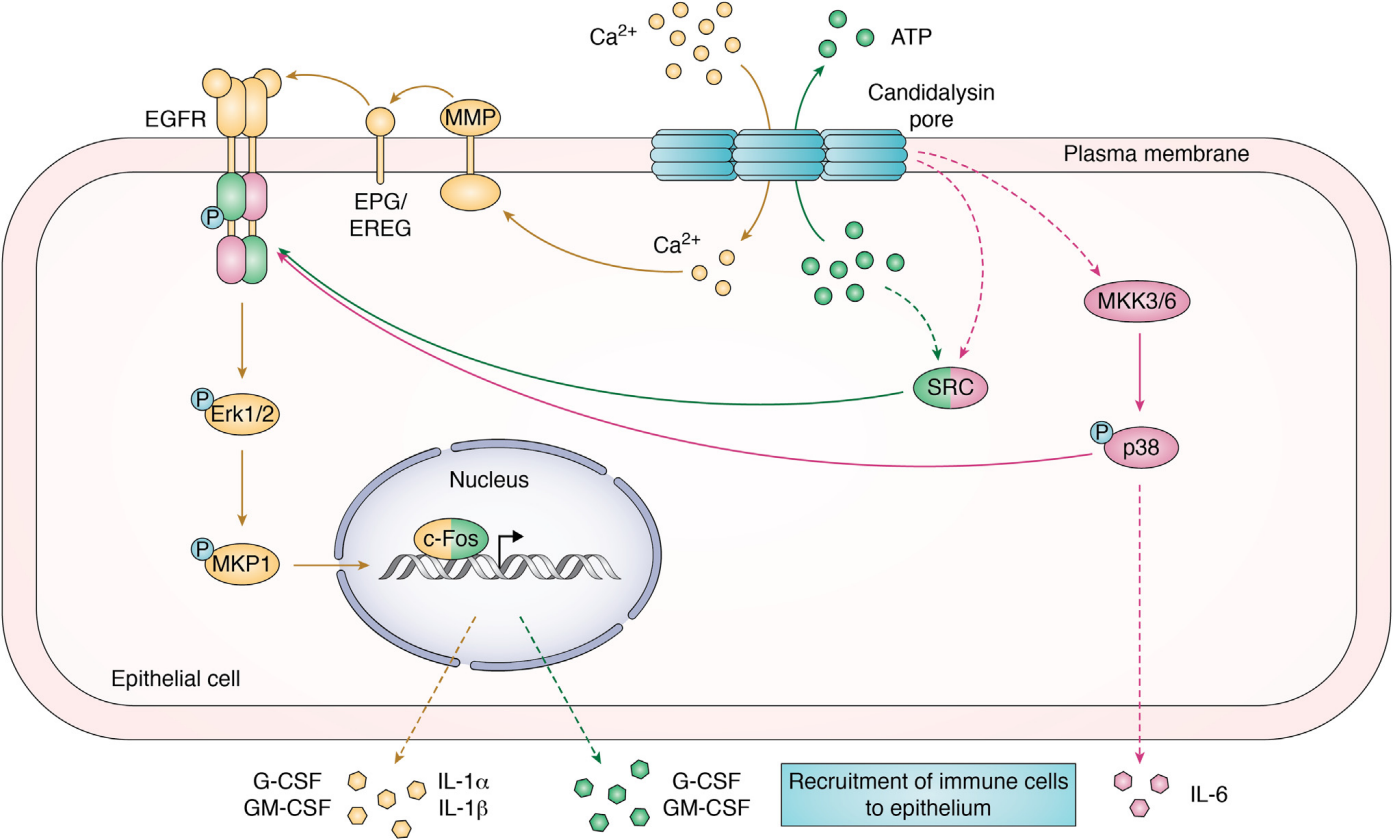
**CL pores promote immune recruitment through multiple pathways.** Each pathway is depicted in a different color, with *solid arrows* indicating direct regulation, and *dashed arrows* referring to indirect or unconfirmed regulation. Proteins involved in more than on pathway are shown with multiple colors. CL pore formation causes ATP efflux (*green*) and calcium influx (*yellow*). ATP efflux activates Src, which partially stimulates EGFR in a ligand-independent manner. At the same time, calcium influx activates MMPs, which cleave EGFR ligands that directly activate EGFR. Fully activated EGFR stimulates the ERK1/2 and MKP1 signaling cascade through phosphorylation, driving downstream activation of c-Fos. c-Fos then promotes secretion of IL-1α, IL-1β, G-CSF, and GM-CSF. Finally, through a mechanism yet unknown (*magenta*), CL activates Src and MKK3/6 to activate p38, which causes the release of IL-6. Release of IL-6, IL-1α, IL-1β, G-CSF, and GM-CSF contributes to recruitment of immune cells to the epithelium. CL, candidalysin; EGFR, epidermal growth factor receptor; IL, interleukin; MMP, matrix metalloproteinase.

To understand how CL activates different signaling pathways, one needs to consider its membrane damaging characteristics. We recently demonstrated that CL functions neither like a detergent, nor similarly to the typical mechanism of membrane active peptides (MAPs), in which binding to the target lipid membrane precedes self-assembly and pore formation (15). CL displays a surprising ability to self-assemble in aqueous solution, where it forms complexes of moderate to large size. To damage membranes, CL self-assembles into polymeric structures that first grow and then close into loops. CL loops bind and insert into the membrane to form pores. We established that CL oligomerization is in fact a requirement for pore formation by identifying a loss-of-function variant of CL (G4W). These *in vitro* structural effects were recapitulated in TR146 oral epithelial cells, further confirming the utility of self-assembly outside of the membrane as part of the mechanism that CL uses to damage human cells (15).

Recently, distinct CL peptides were identified and characterized in two other *Candida* species and amongst *C. albicans* clinical isolates, revealing that CL is indeed a family of cytolytic fungal peptide toxins (16, 17). CL is therefore both the first in a family of fungal peptide toxins, and in a new group of MAPs characterized by self-assembly prior to membrane binding. Excellent reviews describe the signaling cascades that CL induces (3, 18, 19, 20) and the pathologies that CL contributes to (2, 18, 21). In this review, we additionally connect the mechanism CL utilizes to form pores in the plasma membrane with its immunostimulatory properties.

## CL activates damage response signaling pathways

The epithelial response to *C. albicans* is characterized by the activation of NF-κB signaling and a biphasic MAPK mechanism (22, 23). The first phase, coined the epithelial “danger response”, occurs during the recognition of *C. albicans* regardless of morphology. The commensal form activates NF-κB and the first MAPK response through interactions with fungal cell wall structures, including chitin, mannans, and β-glucans, as well as the *C. albicans* invasin Als3. During invasion, Als3 forms complexes with the human proteins E-cadherin, epidermal growth factor receptor (EGFR), HER2, and EphA2, and these complexes simultaneously work to induce endocytosis of the *C. albicans* hyphae (10, 24, 25). However, this initial phase of recognition and endocytosis alone is not sufficient to induce cytokine production and the resulting immune activation (23).

To induce immune activation, *C. albicans* first creates an “invasion pocket” achieved by both hypha-driven physical- and endocytic-mediated plasma membrane invagination. The extending hyphae release CL into the invasion pocket and CL induces the second phase of the MAPK response (4). In this phase, CL activates the EGFR, which leads to phosphorylation of MKP1 and causes activation of the transcription factor c-Fos (12) (Fig. 1). This “damage response” induced by CL releases the proinflammatory cytokines IL-6, IL-1α, IL-1β, G-CSF, and GM-CSF (4). Cytokine secretion causes recruitment of immune cells, including neutrophils and macrophages (26, 27). In some pathologies, immune cell recruitment is essential for fighting pathogenic *C. albicans* (4, 28, 29), while in other cases, the immune response causes disease symptoms (14, 30). Due to this complexity, it is important to continue to advance our understanding of the signaling pathways that regulate the interactions between *C. albicans* and epithelial cells.

A critical gap in our understanding of *C. albicans* infection was the mechanism that CL used to cause epithelial cell inflammatory responses. Initial discovery of CL revealed that low micromolar concentrations of the peptide are enough to induce activation of c-Fos and secretion of the cytokines G-CSF and GM-CSF (4). However, it is not until high levels of CL are present that IL-1α, IL-1β, and IL-6 are released (4). Such differential responses seem to provide a means for the cell to adjust its response to varying doses of CL on its plasma membrane. The differences in cytokine release are likely attributed to the branching signaling pathways that CL activates. One such pathway begins with CL pores allowing an influx of calcium into the cytoplasm (Fig. 1, yellow) (12). Calcium influx activates matrix metalloproteinases, and these proteases cleave free the EGFR ligands epiregulin (EREG) and epigen (EPG). These ligands bind to and activate EGFR to stimulate ERK1/2. ERK1/2 phosphorylates MKP1, and this event induces expression of the transcription factor c-Fos, leading to secretion of G-CSF and GM-CSF. However, activation of EGFR by these two ligands is not sufficient to fully activate c-Fos, suggesting an additional mechanism besides ligand-mediated EGFR activation. In addition, calcium influx does not cause secretion of IL-6 (12), additionally suggesting that calcium is not the sole regulator of inflammatory signals.

While calcium influx causes partial activation of EGFR, an additional CL effect is required for full EGFR activation. Namely, cytoplasmic ATP leaks through CL pores into the extracellular medium (Fig. 1, green) (31). Although the mechanism of ATP-mediated EGFR activation has not been described in the context of CL, another study shows that ATP leakage regulates EGFR activity through DUOX1, which oxidatively activates ADAM17 and the kinase Src (32). ADAM17 is another matrix metalloproteinases that releases EGFR ligands, while Src activates EGFR in a ligand-independent manner (33). The Naglik group showed that chemical or enzymatic depletion of ATP reduces the EGFR-mediated responses of c-Fos, pMKP1, G-CSF, GM-CSF, and IL-6 but does not affect IL-1α or IL-1β (31). Therefore, full activation of EGFR by CL requires simultaneous ligand-dependent and ligand-independent EGFR activation, which are triggered by calcium influx and ATP release, respectively.

As previously stated, calcium influx does not cause the release of IL-6 (12). Recently, it was proposed that the MAPK p38 acts upstream of EGFR to both regulate EGFR activity and IL-6 secretion in a side pathway that is independent of EGFR (Fig. 1, magenta) (34). To do this, CL activates Src and MKK3/6 by mechanisms yet unknown (34), though Src activation for this purpose may be part of the abovementioned ATP pathway (31, 32). Src causes activation by phosphorylating EGFR and p38, while MKK3/6 only activates p38 (34). Both Src and MKK3/6 are required for full CL-mediated activation of p38 (34). Active p38 phosphorylates EGFR, yet the activation of EGFR by p38 alone is not enough to send signals for c-Fos activation (34). p38 also is the main driver for release of IL-6 independent of EGFR activation (34), though the full mechanism of this process is still not clearly understood.

It appears that activation of EGFR by calcium, ATP, and p38-driven pathways are all required for a full inflammatory response to CL in epithelial cells (Fig. 1). It was also recently demonstrated that calcium influx caused by CL induces ESCRT recruitment and lysosome exocytosis. This anti-CL response excises plasma membrane patches rich in CL pores as a mechanism to repair cellular membrane after damage (35). It would not be surprising if the effect of CL on immune signaling goes beyond the scope of the signaling axis described. The fact that there are multiple ways by which EGFR is activated by CL appears to be a form of signal transduction redundancy. However, since no one mechanism causes release of all associated cytokines, it is likely that phosphorylation of specific Tyr residues in the receptor cause differential regulation of downstream signaling. Regardless, EGFR does appear to be the common thread in almost every pathway currently known to regulate CL-induced inflammation. For this reason, EGFR is a promising therapeutic target to alleviate candidiasis symptoms. Indeed, in mouse models of OPC, EGFR inhibitors have been shown to reduce fungal load (12, 25). However, in a zebrafish swim bladder model, EGFR inhibition greatly increased morbidity (12). The opposite responses to EGFR inhibitors likely are a consequence of disparate anatomical sites where *C. albicans* causes disease, which will be discussed in the next section.

## CL directly influences mucosal and systemic pathologies

Through the use of both synthetic peptides and *C. albicans* deletion mutants, CL has now been demonstrated to play significant roles in driving disease pathogenesis in cell culture and animal models at virtually all major biological sites of infection, underscoring its key function as a *bona fide* fungal virulence determinant. While effector mechanisms and protective responses differ between anatomical sites, CL-driven pathology first requires intimate adherence and invasion of *C. albicans* mediated by hyphal growth during active infection (10). CL accumulates at moderate concentrations in the invasion pocket where it can rupture plasma membranes leading to necrosis and activation of specific cellular receptors, as delineated in the prior section, to elicit immunological signal transduction (at lower CL levels) (8, 36). Necrosis and general cellular toxicity are typically measured by LDH release. It was initially described that high CL concentrations (>70 μM) are necessary to induce significant LDH release and subsequent cell death (4, 8). However, it is well established that low concentrations of CL can activate immune signaling in epithelial cells in multiple pathologies. Below we discuss the impact of CL during both mucosal and invasive candidiasis.

### Oropharyngeal candidiasis

Susceptibility to oral candidiasis is largely driven by genetic mutations, comorbid infections (*e.g.*, HIV), or transient immunosuppression, resulting in invasion of oral and pharyngeal epithelial tissue by *C. albicans* that causes localized, painful inflammation. In fact, the mouse model of OPC was the first to demonstrate a critical role for CL in driving mucosal pathogenicity (37, 38). Moyes *et al.* (4) showed that *C. albicans* mutants harboring full-length *ECE1* or CL-specific deletions colonized epithelial tissue less robustly than a wildtype (WT) strain and elicited less proinflammatory cytokines (including IL-1⍺, G-CSF, GM-CSF, and IL-6), neutrophil recruitment, and tissue damage when infecting the murine oral cavity. Similar studies (4, 29, 34, 39) performed on TR146 oral epithelial cells using the aforementioned strains and CL peptide demonstrated that these effectors were largely driven *via* activation of the c-Fos transcription factor downstream of MAPK signaling pathways. However, tissue damage was driven by generally high levels of CL, suggesting roles for both lytic and nonlytic mechanisms of immune induction.

Both conventional CD4+ and γ/δ T-cells are essential for protection against oral candidiasis, as their depletion renders mice and humans hypersusceptible to *C. albicans* colonization and tissue invasion (40). The Gaffen laboratory has further shown that innate or “natural” IL-17 responses to *C. albicans* are a protective lynchpin, as IL-17RA−/− mice that cannot sense IL-17 isoforms phenocopy chemotherapeutically immunosuppressed mice rendered defective in T-cell and neutrophil responses during OPC (41, 42). Follow-up studies revealed that CL drives expansion of innate IL17+TCR⍺β+ cells *via* IL-1 signaling, which orchestrate protective responses at the oral mucosa *via* production of IL-1R-dependent effectors, including defensins, alarmins, and neutrophil chemoattractants (39). CL and IL-17 synergize to further enhance antifungal host defenses in a feed forward activation loop. IL-22 is another major effector of the IL-17 signaling axis, and its expression is similarly reduced in oral epithelium during challenge with an *ece1*Δ/Δ or CL deletion strain as compared to infection with the WT strain (43). Induction of IL-22 activates the transcription factor STAT3 in basal epithelial layers to drive an IL-17–specific gene signature that replenishes IL-17RA-expressing suprabasal epithelial layers to continue responding to IL-17 signals that further enhance antifungal effector responses. Aside from IL-17-dependent responses, key roles for CL in activating EGFR and IL-36 signaling in driving protection and immune activation during oral candidiasis have also recently been established (12, 13).

### Vulvovaginal candidiasis

Unlike OPC, which results from immunosuppression, VVC is a disease of immunocompetent and otherwise healthy women (44). Overgrowth of *C. albicans* in the vagina precipitates onset of disease symptoms, including burning and itching. Use of high estrogen-containing contraceptives, antibiotic use, and sexual activity are associated with increased risk of infection. However, the precise mechanisms that govern disease susceptibility and pathogenesis are poorly understood (45). The prevailing hypothesis states that VVC is an immunopathology, by which strong proinflammatory signals generated at the vaginal mucosa by *C. albicans* and subsequent neutrophil recruitment are not only insufficient to clear the infection but instead drive disease symptoms (30).

**Figure 2:**
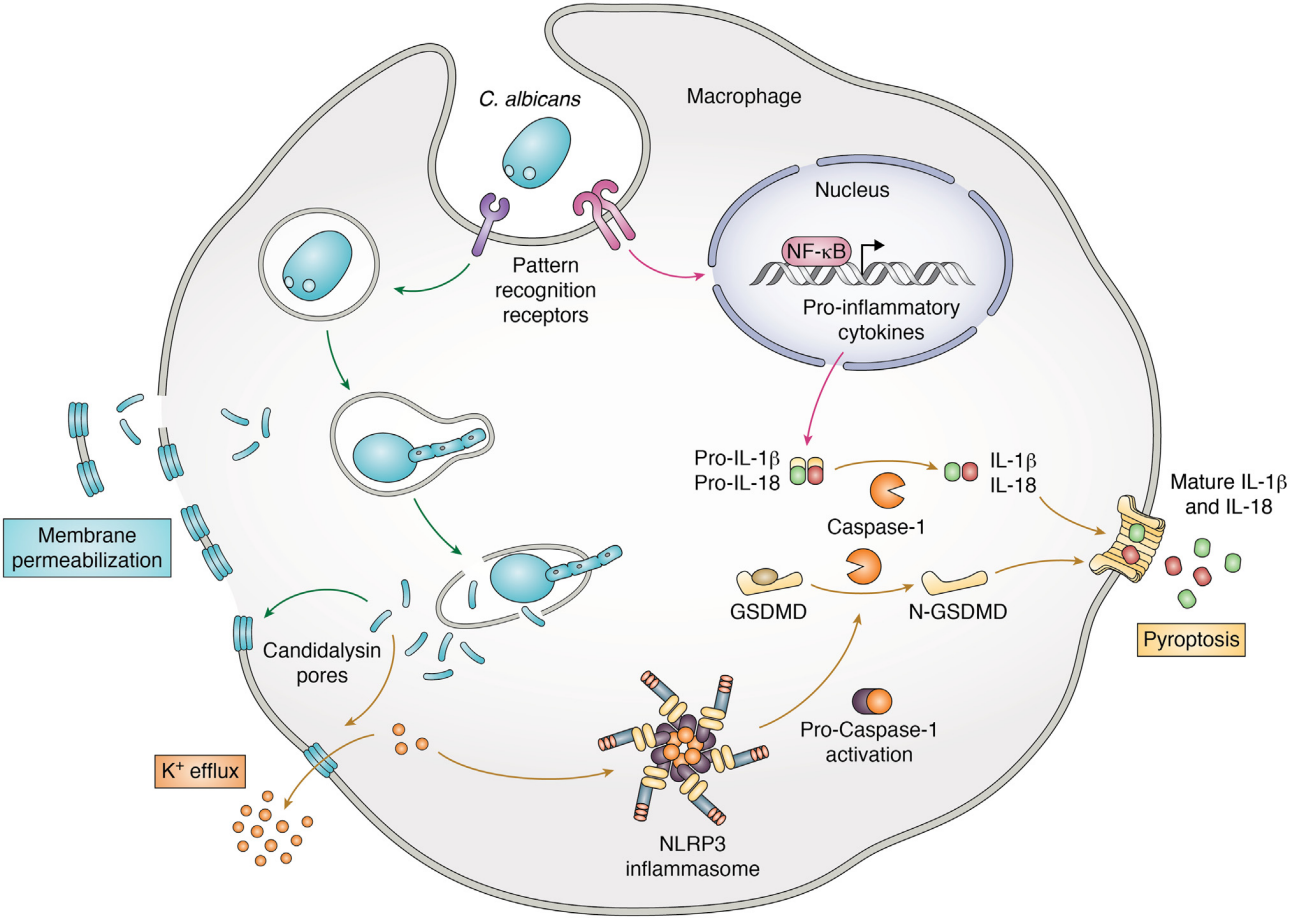
**CL damages macrophages and induces inflammasome activation.** Pattern recognition receptors (PRRs) on macrophages are used to trigger *C. albicans* phagocytosis. Inside the phagosome, *C. albicans* forms hyphae and secretes CL—both of which facilitate macrophage escape. Additionally, CL intercalates into the plasma membrane and causes bilayer rupture and K^+^ efflux. The release of this ion activates the NLRP3 inflammasome and subsequently caspase-1, which produces Gasdermin-D (GSDMD) and mature IL-1β and IL-18. These cytokines then traverse the membrane through GSDMD pores to drive pyroptosis. CL, candidalysin; IL, interleukin.

Challenge with *ece1*Δ/Δ or CL deletion mutants in the murine model of VVC resulted in reduced secretion of proinflammatory cytokines, severely attenuated neutrophil recruitment to the vaginal lumen and decreased tissue damage as compared to challenge with a WT strain (14). These mutants still robustly formed hyphae in the vaginal lumen, demonstrating that hyphal growth is necessary but not sufficient for VVC immunopathology and that CL is the key virulence determinant in driving these responses. Moreover, synthetic CL was sufficient to elicit similar cytokines and damage in human vaginal epithelial cells. Despite the clear protective role for IL-17 in the oral cavity, no such role has been established during VVC (46, 47). Instead, work in recent years has implicated the NLRP3 inflammasome (Fig. 2), a danger sensing immune complex, in governing characteristic neutrophil recruitment during human and experimental vaginal candidiasis, as NLRP3−/− mice exhibit reduced immunopathology during infection (48, 49, 50). The intracellular sensor NLRP3 can oligomerize, activate the protease Caspase-1, and ultimately secrete the major inflammasome effector IL-1β upon sensing cell damage through a variety of mechanisms (51). Consistent with activity of other microbial toxins, CL was shown to be both necessary and sufficient for activating NLRP3 and driving IL-1β release in both murine bone marrow–derived macrophages and human THP1 macrophage-like cell lines, primarily *via* a mechanism involving potassium efflux (52, 53, 54).

Interestingly, clinical isolates of *C. albicans* from human subjects with acute VVC are correlated with high *ECE1* expression, as compared to isolates derived from asymptomatic carriers. This agreement further strengthens the observed connection between inflammasome signaling and symptomatic disease (55). More recent work has shown that additional CL isoforms exist among vaginal clinical isolates and that at least one of these is associated with reduced neutrophil recruitment during murine VVC due to reduced capacity to be processed and secreted by *C. albicans* (17). Additionally, other CL isoforms exist in the non-*albicans Candida* species *C. dubliniensis* and *C. tropicalis*. While these isoforms demonstrate exaggerated cytolytic and immunostimulatory effects as synthetic toxins (see below), they are expressed less robustly at the transcriptional level in these non-*albicans Candida* species as compared to the *C. albicans* reference isolate SC5314 (17). Therefore, these collective results suggest that CL expression and activity varies widely across the population and may partly explain disparate vaginopathogenicity among isolates.

### Gastrointestinal candidiasis

Gastrointestinal (GI) candidiasis results from fungal overgrowth at the esophageal or intestinal mucosa. The onset of GI candidiasis is most commonly initiated by depletion of protective neutrophils during chemotherapy (upper GI tract) or of beneficial microbiota during extended, high-dose antibiotic therapy (lower GI tract) (56). Ulceration and erosion of the esophageal and intestinal lining ensues, leading to inflammation driven by imbalanced T-reg, Th1, and Th17 T-cell effector responses (57). Exacerbated inflammatory responses then further drive higher fungal colonization (58). While CL has been strongly implicated in driving the pathogenesis of OPC and VVC, its role in contributing to GI candidiasis is less clear. This is partly due to the fact that *C. albicans* traditionally adopts yeast or GUT (gastrointestinally induced transition) morphologies during colonization of the murine GI tract (59). As CL is regarded as hypha-specific, this suggests that CL does not play a major role during GI infection. However, subpopulations of colonizing *C. albicans* may adopt hyphal morphologies that can express and secrete CL. The importance of this possibility was demonstrated by Allert *et al.* (60) who showed that CL was essential for invasion, damage of, and translocation across enterocyte monolayers. As the GI tract is a major reservoir for *C. albicans* in the human host and an endogenous source of hematogenous spread in the face of immunosuppression, even infrequent breach of epithelial layers may be of significant importance (61, 62). Further supporting this notion, mycobiota sequencing has revealed that a rich diversity of *C. albicans* strains dominate the colonic mucosa of human subjects with inflammatory bowel disease, characterized by inflamed colonic epithelium (63). Fungal isolates that exhibited the capacity to strongly elicit IL-1β release by macrophages were correlated with worse inflammatory bowel disease severity and elevated CL expression. While this was not shown to be explicitly NLRP3 dependent, it is reasonable to speculate given that one study has demonstrated hyperactivation of NLRP3 in Crohn’s Disease patients (60%) as compared to healthy controls (28.6%) (64). Similar to vaginal isolates, GI isolates exhibit phenotypic heterogeneity with respect to growth, yeast-to-hypha transition, and *ECE1* expression, underscoring that isolate-to-isolate diversity impacts disease outcome (65). Therefore, CL may push or exacerbate GI dysbiosis and precipitate immunopathogenesis of the human gut.

### Systemic candidiasis

Systemic candidiasis results from failure of innate biological barriers, primarily *via* breaches of the GI tract or use of indwelling medical devices (*e.g.*, intravenous catheters), which allow *C. albicans* to freely disseminate *via* the bloodstream to seed body organs and tissues where localized sequalae develop. Diminished immune surveillance at biological barriers also contributes to disseminated disease (66). While CL clearly facilitates invasion of mucosal barriers, until recently, it was unclear whether it would play an important role once these are bypassed. In order to answer this question, Swidergall *et al.* (29) utilized the murine model of invasive candidiasis by injecting WT or *ece1*Δ/Δ *C. albicans* directly into the lateral tail vein of immunocompetent mice. Remarkably, mice challenged with the WT strain developed a strong early inflammatory response (also recapitulated in human endothelial cells) characterized by robust neutrophil recruitment and elevated lethality. Challenge with the *ece1*Δ/Δ mutant resulted in reduced early cytokine production and neutrophil recruitment and failure to control fungal burden in systemic organs (kidney, brain, and spleen). Depletion of neutrophils rendered both strains equally virulent, confirming that CL serves dual roles as a driver of pathogenicity but also in sounding the alarm for infection control. Work by Drummond *et al.* (28) showed that CL-dependent IL-1β activation by brain microglia recruit neutrophils to this organ to control fungal burden and likely explain the high levels of brain colonization observed by Swidergall *et al.* Collectively, these studies demonstrate that CL serves important roles beyond the epithelial and endothelial interface.

## CL uses a novel mechanism of pore formation to damage human cells

**Figure 3:**
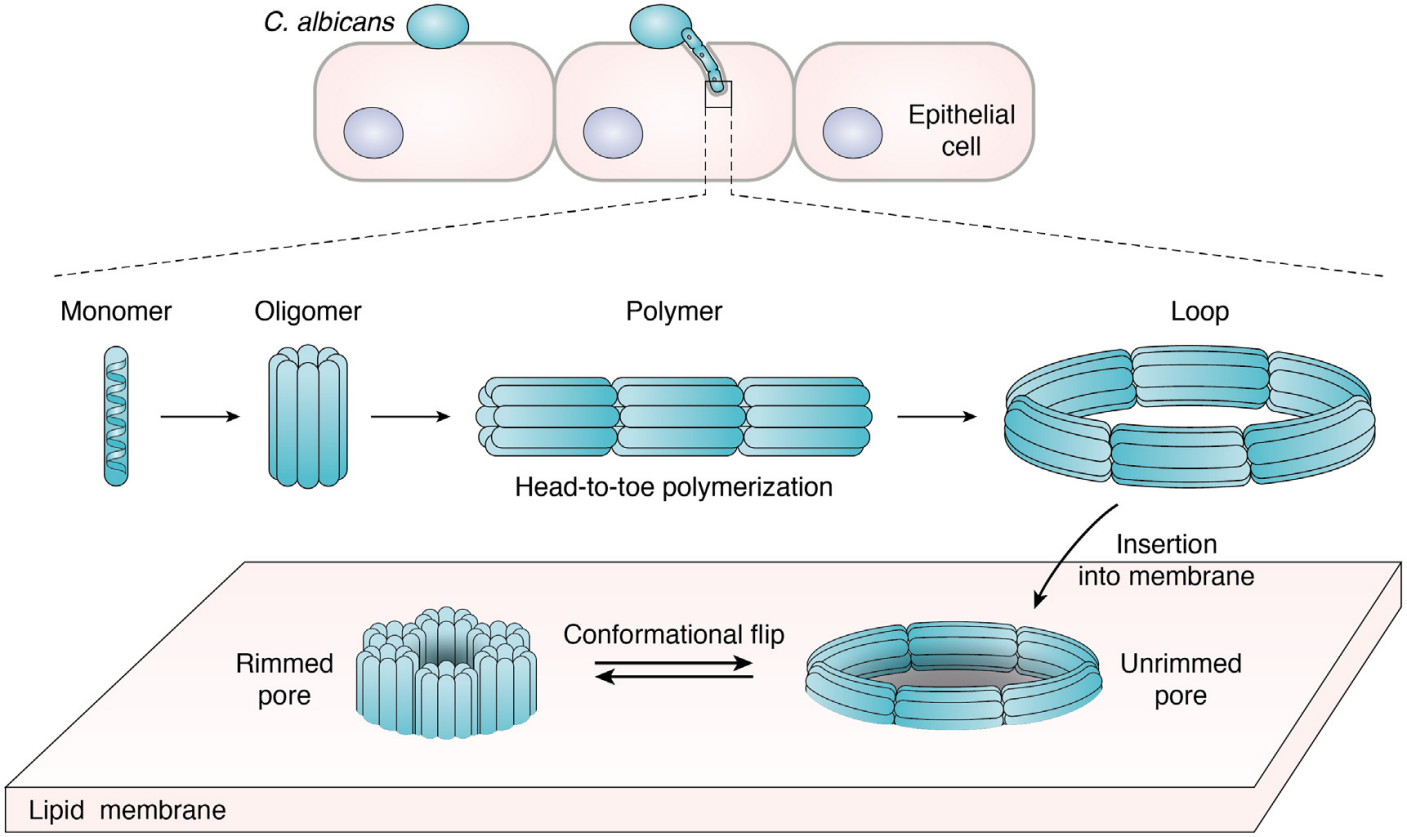
**Candidalysin undergoes complex self-assembly before binding to the plasma membrane, where it forms pores.***C. albicans* hyphae form an invasion pocket in epithelial cells. Candidalysin oligomerizes outside of the plasma membrane. Oligomers polymerize head-to-toe into long chains that close to form membrane-competent loops. Loops insert into epithelial cell membranes probably as unrimmed pores. A conformational flip of CL oligomers into a vertical orientation triggers the rimmed pore conformation and presumedly increases cellular toxicity. CL, candidalysin.

We are beginning to understand the immunopathology that CL elicits through membrane disruption and subsequent activation of signaling cascades (10, 11, 14, 34). However, until recently, we lacked a clear mechanistic understanding of precisely how CL physically damages cell membranes. Initial hypotheses about how CL permeabilizes membranes relied on prior understanding of MAPs. MAPs such as melittin, alamethicin, and LL-37 are peptides produced by their hosts to damage target plasma membranes (67, 68, 69, 70, 71). MAPs typically use a mechanism of membrane disruption in which binding to the target lipid membrane is required for peptide self-assembly. Moyes *et al.* (4) biophysically showed that CL interacts with synthetic bilayers. Noting this phenomenon and the sequence similarities CL has to other MAPs (69, 71), it was hypothesized that CL perforates cell membranes using a “carpet-like” mechanism due to the occurrence of calcium influx and LDH release in oral epithelial cells (4). Their experiments in synthetic membranes suggested CL formed a pore, motivating biophysical exploration of its mechanism. To this end, our laboratory recently demonstrated that CL utilizes a novel mechanism of pore formation to damage human oral epithelial cells (Fig. 3) (15).

Prior to the discovery of CL, it was accepted that MAPs cause membrane damage through a common mechanism in which secreted, monomeric peptides must bind to the target cell membrane to oligomerize and form a pore (68, 71). The intermediate steps vary by peptide, but membrane interactions are required for self-assembly. Strikingly, we observed that CL readily self-assembles in the absence of a lipid membrane in polymeric chains (15) formed by sequential linkage of an oligomer, which we propose is an octamer. CL oligomers assemble head-to-toe, as opposed to side-by-side, and once the polymeric chains of oligomers grow to a certain length, they close to form loops.

**Figure 4:**
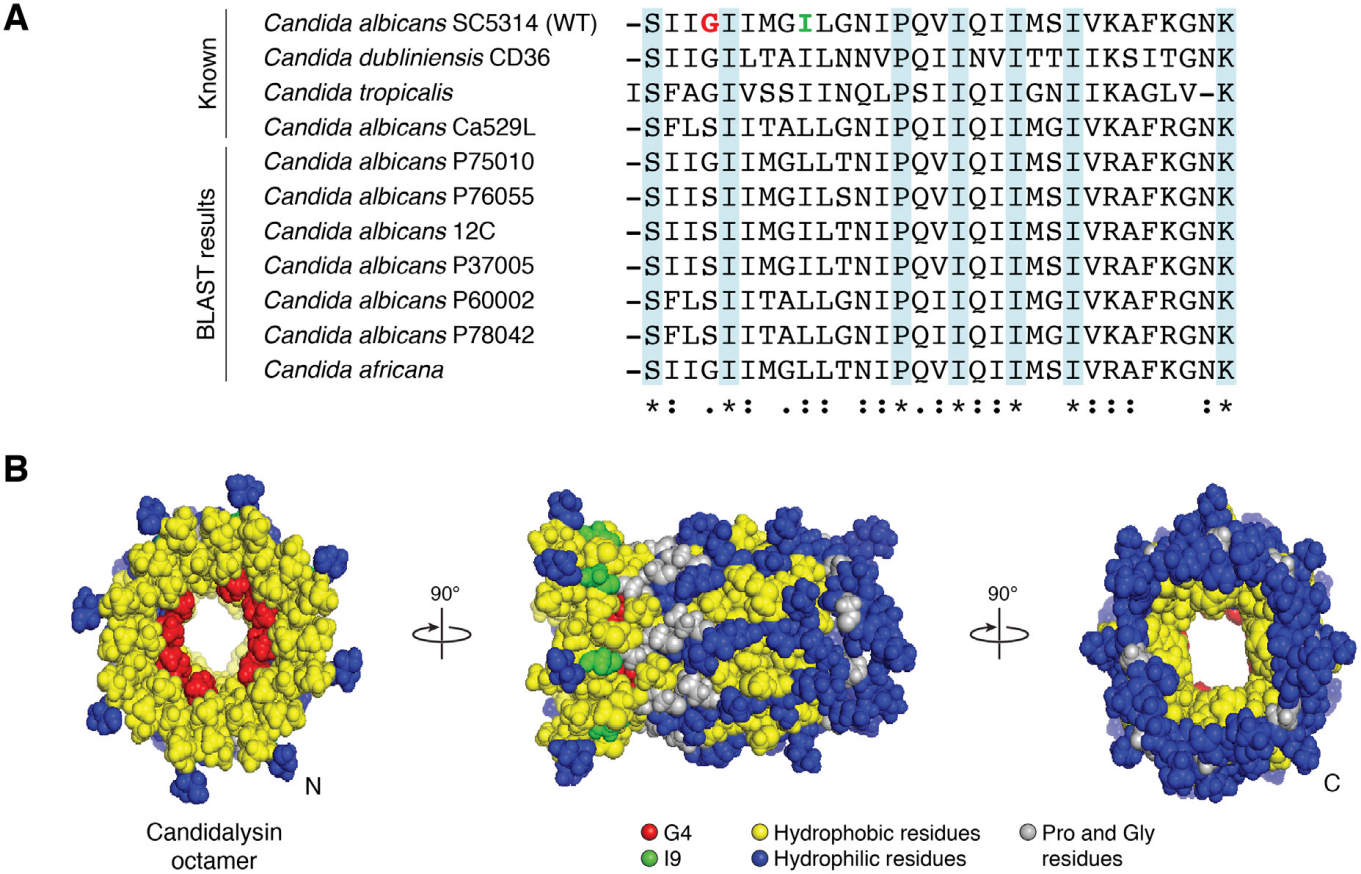
**CL sequence variation.***A*, table contains known CL sequences, and BLAST results of additional potential CL among *Candida* species. G4 (*red*) and I9 (*green*) residues are highlighted in the WT sequence and in the proposed oligomer (octamer) model. *B*, octamer shown in three orientations. Hydrophobic residues are colored *yellow*, and hydrophilic residues are shown in *blue*. Pro and other Gly residues are shown in *gray*. Model generated in PyMOL. CL, candidalysin.

We further investigated the interaction between CL and membrane lipids. Though we only observed one type of loop structure in solution, using atomic force microscopy, the laboratory of Gavin King identified two different types of pore structures that differed in size and membrane topology. One type of pore had a similar diameter to the loops observed in solution. This pore has the same thickness as the lipid membrane, and we termed these “unrimmed pores.” In contrast, we observed slightly smaller pores with elevated features around the perimeter, which we in turn named “rimmed pores”. Our data suggest that these two populations represent an uncharacterized maturation mechanism resulting from a conformational rearrangement of CL oligomers that occurs after insertion into the lipid bilayer (Fig. 3). In brief, our model posits that the loops formed in solution can insert into the membrane forming unrimmed pores. Since in this type of pore CL oligomers still interact in a head-to-toe fashion, all the positively charged residues, which are present at the C terminus (C_t_) of the sequence and the charged N-terminal (N_t_) group (Fig. 4), will be at the hydrophobic core of the membrane. The presence of charges in the membrane bilayer is thermodynamically unfavorable; as a result, the unrimmed pore arrangement is bound to be unstable. We hypothesize that repositioning of the charged residues encourages the oligomers to be rearranged into the rimmed pore configuration, where the charged N_t_ and C_t_ are hydrated at either side of the bilayer. This would additionally allow for the positively charged Lys residues to interact with anionic lipids present in the inner leaflet of the plasma membrane to further stabilize the rimmed pore conformation. Since we have observed that rimmed pores are stable for longer periods of time, we therefore propose that they cause the most effective membrane damage (15). However, this mechanism of pore stabilization and maturation is still uncharacterized and requires further study.

We further elucidated the connection between CL self-assembly and pore formation using gain-of-function (I9A) and loss-of-function (G4W) variants (15). *In vitro*, I9A formed more abundant populations of loop structures in comparison to WT, while G4W did not efficiently self-assemble. Changes in self-assembly competence directly correlated to membrane damage, as I9A exhibited increased membrane damage while G4W was unable to damage lipid vesicles. Furthermore, pore formation *in vitro* was a good predictor of damage signaling in the oral epithelial cell line TR146. G4W was unable to activate c-Fos, a key player in the damage signaling cascade whereas I9A effectively increased expression. These results indicated that self-assembly in solution is a prerequisite to pore formation (15). This process represents a novel mechanism of pore formation and importantly identifies CL self-assembly as a novel potential therapeutic target.

## The CL family of fungal peptide toxins

Since the discovery of CL as the first known pathogenic cytolytic fungal peptide, others in this family have been discovered (16, 17). Two variants of CL were recently identified in other species of *Candida* that contribute to the progression of invasive candidiasis, *C. dubliniensis* and *C. tropicalis*. Richardson *et al.* (16) characterized the CL peptides secreted by *C. dubliniensis* and *C. tropicalis* (CL_dub_ and CL_trop_, respectively) and compared them to the CL produced by the original *C. albicans* isolate (CL_alb-SC5314_). In addition, an isoform was identified from the oral isolate 529L (CL_alb-529L_). We found that this isoform exhibited reduced pathogenicity when introduced into an *ece1*Δ/Δ mutant as compared to the original isolate (17). All CL peptides are generated from cleavage of the polypeptide Ece1p at flanking KR sites. Although a synthetic CL_alb-529L_ peptide was capable of driving similar damage and immune stimulation in vaginal epithelial cells as CL_alb-SC5314_, amino acid changes flanking the KR cleavage site negatively impacted secretion (17). Thus, the entire Ece1p polypeptide may also dictate pathogenicity. While all CLs disrupt synthetic lipid bilayers and activate signaling in epithelial cells, synthetic CL_dub_ and CL_trop_ are more toxic than CL_alb-SC5314_, despite their species of yeast being generally less pathogenic. This apparent paradox can be explained because CL_dub_ and CL_trop_ are less transcriptionally abundant and therefore are presumed to be released at lower levels.

Considering the I9A and G4W mutations, as well as the natural *C. albicans* CL variants, it is clear that small sequence changes in CL can impact membrane damage and pathogenic properties. This motivated us to perform a BLAST search for CL sequences in other *Candida* species. Our results, shown in Figure 4*A*, additionally identify CL sequences with striking sequence similarity in different strains of *C. albicans* as well as in other *Candida* species—*C. africana* and possibly *C. maltosa* (not shown). Many *C. albicans* strains produce a CL with the same sequence as our WT strain SC5314 (those were excluded from the table). Moreover, our prior sequencing of a collection of *C. albicans* clinical isolates revealed that CL isoforms generally fall into CL_alb-SC5314_- or CL_alb-529L_-like sequences (54). Analyzing these sequences in tandem with our mutational analysis of the SC5314 sequence provides new insights into the function of different residues within CL.

CL forms an amphipathic helix, where hydrophobic residues primarily align into a single helical surface (Fig. 4*B*, yellow). The model generated for the CL oligomer, which we propose is an octamer (15), resembles a hollow cylinder where all hydrophilic residues (blue) face the outer surface. This circumstance could explain the high solubility of CL in water. The hydrophobic residues (yellow) preferentially form the helix–helix interface, constituting a central core in the oligomer. It seems likely that this arrangement would change once CL binds to the lipid membranes; indeed, we have proposed that this binding event could trigger a conformational change that transforms unrimmed pores into the rimmed counterparts. After a hypothetical rotation of the helices within the oligomer, hydrophobic residues would interact with the surrounding lipids, while polar residues would reorient to form a polar core in the oligomer.

Only seven out of the 31 CL residues are totally conserved. Two of those are the terminal residues, Ser (at the N_t_) and Lys (at the C_t_). This last Lys is conserved out of evolutionary constraints, as it is required by the specificity of Kex proteases. Additionally, four central Ile residues are conserved and the only Pro. Prolines destabilize helical structures and are commonly observed in central positions of MAPs and other membrane-interacting peptides (67, 69, 72, 73). This residue possibly prevents uncontrolled aggregation and aids in membrane insertion and stabilization.

The first few residues at both the N_t_ and C_t_ ends of any α-helix are typically unstructured due to the lack of stabilizing hydrogen bonds, in what is known as helix fraying (74, 75, 76). It is possible that the unstructured N_t_ of a CL oligomer constitutes a flexible binding site for the unstructured C_t_ of an opposing oligomer. This arrangement would be electrostatically stabilized by interaction between the positively charged terminal N_t_ amine with the negatively charged C_t_ carboxyl group. However, additional interactions that are not general to any polypeptide are needed to provide specificity. We ponder that the conserved N_t_ Ser could form hydrogen bonds with the C_t_ Asn or Lys, contributing to a fuzzy interaction interface. It is interesting to observe that the G4S mutation is common among CLs (Fig. 4*A*). The underlying reason might be that the Ser residue, while less destabilizing than Gly, still allows flexibility. This would lend support to the hypothesis that CL polymerizes through interaction of the unstructured ends of the sequence. Such flexibility seems functionally relevant, since electron microscopy and atomic force microscopy data show that CL polymers are remarkably flexible (15), and this property likely allows efficient closing of the linear polymer into a ring.

Our mutational analysis (15) provided specific insight into the roles that certain residues play in its function. As previously discussed, the G4W loss-of-function mutant reduces the ability to self-assemble into a pore-competent structure. Replacement of G4 with the W residue confers rigidity to the N_t_. The behavior of G4W suggests the importance of maintaining a flexible N_t_, possibly because of its ability to allow self-assembly of oligomers into a head-to-toe alignment. The I9A gain-of-function variant displays enhanced self-assembly and membrane-damaging abilities. I9 is one of several Ile residues scattered throughout the N_t_. Specifically, I9 extends into the lumen of our oligomer model (Fig. 3*B*). We propose that replacing I9 with a smaller Ala residue reduces steric hindrance between branched amino acids, allowing for more efficient oligomerization and therefore pore formation. The outer surface of a CL octamer contains several N_t_ hydrophobic amino acid residues with long, aliphatic chains facing outward into the aqueous solution. This orientation would be thermodynamically unfavorable, encouraging interaction with the plasma membrane of target human epithelial cells. Studying I9A and G4W demonstrated how small alterations in the primary sequence can cause important functional changes.

We observed that *C. albicans* CL forms two types of pores, unrimmed and rimmed. We hypothesize that rimmed pores are formed from an uncharacterized maturation mechanism in which CL oligomers aligned parallel to perpendicular to the membrane plane flip ∼90 degrees. This conformational change is likely stabilized by electrostatic attraction between the cationic C_t_ residues and anionic lipids present at the inner leaflet of the plasma membrane. It is likely that CL additionally utilizes a N_t_ hydrophobic interaction to facilitate initial binding with the membrane post polymerization. Additionally, the C_t_ GNK motif is found in almost every CL variant. Perhaps this sequence plays an additional role in pore stabilization postinsertion.

## Conclusion

CL is a peptide toxin that perforates cell membranes which follows a route never observed before. This unique mechanism of pore formation is expected to influence the complex signaling that CL triggers. It is well established that EGFR is a central keystone of CL immune signaling in epithelial cells. However, as shown in Figure 1, it is also clear that the signaling that CL induces extends beyond EGFR activation. The p38 pathway, which is independent of EGFR, is necessary for IL-6 release, although we still do not understand the intermediate steps that lead to secretion of this cytokine. The other two established signaling pathways that CL activates also result from pore formation, allowing for the translocation of calcium and ATP in and out of the cytoplasm, respectively. Whether signaling and response dynamics are conserved across other cell types that CL attacks, like macrophages, warrants further investigation.

We recently demonstrated that CL self-assembly is required for both pore formation and immune activation (15). However, it is currently unknown precisely how CL is secreted from *C. albicans* hyphae after cleavage by the Kex proteases. Considering the mechanism of membrane disruption that we have proposed, the oligomeric state in which CL is released is expected to influence its function inside the invasion pocket formed by *C. albicans*. Since CL self-assembles efficiently, is it likely that CL will be released as an oligomer or even as a fully assembled prepore structure. If CL self-assembles presecretion, then this would allow for quicker and more efficient pore formation, but a large structure might be harder to exocytose out of the fungal cell. Furthermore, there are seven other peptides generated from Ece1 with no known function. It seems unlikely that these peptides could simply be waste products of CL generation. CL is not known to damage its own fungal membrane, as this would obviously be counterproductive. Perhaps some of the other Ece1 peptides serve to protect *Candida* from CL self-attack, as it has been observed in other organisms that proteins can act as a “toxin sponge” that prevents self-harm by absorbing the toxic molecules (77). Alternatively, these peptides may prevent premature aggregation before secretion or the Ece1 polypeptide itself could act to hold CL in a nonfunctional state until processed. It is clear that studying these seven other peptides and delineating CL’s secretion mechanism will provide a deeper knowledge of CL biology.

Understanding the mechanism by which CL and its variants damage human cell membranes is key to efficiently treating *Candida* infections. The signaling axis that CL activates through its damaging properties is complex and intertwined. It is likely that other host signals will be identified as necessary during the CL-induced immune response. The ability of a CL pore to cause the release of cytoplasmic materials is a known mechanism of immune activation. However, the physical stresses that these pores induce on the membrane may also have activation consequences. Regardless, these signaling activation mechanisms hinge on the ability of CL to self-assemble into a pore-competent structure. Identifying strategies aimed to prevent CL’s self-assembly would therefore constitute promising therapeutic avenues to treat *Candida* infections.

## Conflict of interest

The authors declare no conflict of interest with the contents of this article.

## References

[1] Mayer F.L., Wilson D., Hube B. (2013). Candida albicans pathogenicity mechanisms. Virulence.

[2] Ho J., Camilli G., Griffiths J.S., Richardson J.P., Kichik N., Naglik J.R. (2021). Candida albicans and candidalysin in inflammatory disorders and cancer. Immunology.

[3] Naglik J.R., Gaffen S.L., Hube B. (2019). Candidalysin: discovery and function in Candida albicans infections. Curr. Opin. Microbiol..

[4] Moyes D.L., Wilson D., Richardson J.P., Mogavero S., Tang S.X., Wernecke J. (2016). Candidalysin is a fungal peptide toxin critical for mucosal infection. Nature.

[5] Richardson J.P., Ho J., Naglik J.R. (2018). Candida–epithelial interactions. J. Fungi (Basel).

[6] Wächtler B., Citiulo F., Jablonowski N., Förster S., Dalle F., Schaller M. (2012). Candida albicans-epithelial interactions: dissecting the roles of active penetration, induced endocytosis and host factors on the infection process. PLoS One.

[7] Willems H.M.E., Lowes D.J., Barker K.S., Palmer G.E., Peters B.M. (2018). Comparative analysis of the capacity of the candida species to elicit vaginal immunopathology. Infect. Immun..

[8] Mogavero S., Sauer F.M., Brunke S., Allert S., Schulz D., Wisgott S. (2021). Candidalysin delivery to the invasion pocket is critical for host epithelial damage induced by Candida albicans. Cell Microbiol..

[9] Richardson J.P., Mogavero S., Moyes D.L., Blagojevic M., Krüger T., Verma A.H. (2018). Processing of Candida albicans Ece1p is critical for Candidalysin maturation and fungal virulence. mBio.

[10] Swidergall M., Solis N.V., Millet N., Huang M.Y., Lin J., Phan Q.T. (2021). Activation of EphA2-EGFR signaling in oral epithelial cells by Candida albicans virulence factors. PLoS Pathog..

[11] Moyes D.L., Murciano C., Runglall M., Islam A., Thavaraj S., Naglik J.R. (2011). Candida albicans yeast and hyphae are discriminated by MAPK signaling in vaginal epithelial cells. PLoS One.

[12] Ho J., Yang X., Nikou S.A., Kichik N., Donkin A., Ponde N.O. (2019). Candidalysin activates innate epithelial immune responses via epidermal growth factor receptor. Nat. Commun..

[13] Verma A.H., Zafar H., Ponde N.O., Hepworth O.W., Sihra D., Aggor F.E.Y. (2018). IL-36 and IL-1/IL-17 drive immunity to oral candidiasis via parallel mechanisms. J. Immunol..

[14] Richardson J.P., Willems H.M.E., Moyes D.L., Shoaie S., Barker K.S., Tan S.L. (2018). Candidalysin drives epithelial signaling, neutrophil recruitment, and immunopathology at the vaginal mucosa. Infect. Immun..

[15] Russell C.M., Schaefer K., Dixson A., Gray A., Pyron R.J., Alves D. (2022). The C. albicans virulence factor candidalysin polymerizes in solution to form membrane pores and damage epithelial cells. Elife.

[16] Richardson J.P., Brown R., Kichik N., Lee S., Priest E., Mogavero S. (2022). Candidalysins are a new family of cytolytic fungal peptide toxins. mBio.

[17] Liu J., Willems H.M.E., Sansevere E.A., Allert S., Barker K.S., Lowes D.J. (2021). A variant ECE1 allele contributes to reduced pathogenicity of Candida albicans during vulvovaginal candidiasis. PLoS Pathog..

[18] Chen Y.-Q., Li Q., Zhang T.-Y., Liu N.-N. (2021). Candidalysin: from mechanism of action to biomarker development and therapeutic response. Innov. Digit. Health Diagn. Biomark..

[19] König A., Hube B., Kasper L. (2020). The dual function of the fungal toxin candidalysin during Candida albicans-macrophage interaction and virulence. Toxins (Basel).

[20] Pellon A., Sadeghi Nasab S.D., Moyes D.L. (2020). New insights in Candida albicans innate immunity at the mucosa: toxins, epithelium, metabolism, and beyond. Front. Cell. Infect. Microbiol..

[21] Li X., Leonardi I., Iliev I.D. (2017). Candidalysin sets off the innate alarm. Sci. Immunol..

[22] Roeder A., Kirschning C.J., Schaller M., Weindl G., Wagner H., Korting H.C. (2004). Induction of nuclear factor-κB and c-Jun/activator protein-1 via toll-like receptor 2 in macrophages by antimycotic-treated Candida albicans. J. Infect. Dis..

[23] Moyes D.L., Runglall M., Murciano C., Shen C., Nayar D., Thavaraj S. (2010). A biphasic innate immune MAPK response discriminates between the yeast and hyphal forms of candida albicans in epithelial cells. Cell Host Microbe.

[24] Phan Q.T., Myers C.L., Fu Y., Sheppard D.C., Yeaman M.R., Welch W.H. (2007). Als3 is a Candida albicans invasin that binds to cadherins and induces endocytosis by host cells. PLoS Biol..

[25] Zhu W., Phan Q.T., Boontheung P., Solis N.V., Loo J.A., Fillera S.G. (2012). EGFR and HER2 receptor kinase signaling mediate epithelial cell invasion by Candida albicans during oropharyngeal infection. Proc. Natl. Acad. Sci. U. S. A..

[26] Erwig L.P., Gow N.A.R. (2016). Interactions of fungal pathogens with phagocytes. Nat. Rev. Microbiol..

[27] Netea M.G., Brown G.D., Kullberg B.J., Gow N.A.R. (2008). An integrated model of the recognition of Candida albicans by the innate immune system. Nat. Rev. Microbiol..

[28] Drummond R.A., Swamydas M., Oikonomou V., Zhai B., Dambuza I.M., Schaefer B.C. (2019). CARD9+ microglia promote antifungal immunity via IL-1β- and CXCL1-mediated neutrophil recruitment. Nat. Immunol..

[29] Swidergall M., Khalaji M., Solis N.V., Moyes D.L., Drummond R.A., Hube B. (2019). Candidalysin is required for neutrophil recruitment and virulence during systemic Candida albicans infection. J. Infect. Dis..

[30] Fidel P.L., Barousse M., Espinosa T., Ficarra M., Sturtevant J., Martin D.H. (2004). An intravaginal live Candida challenge in humans leads to new hypotheses for the immunopathogenesis of vulvovaginal candidiasis. Infect. Immun..

[31] Ho J., Wickramasinghe D.N., Nikou S.A., Hube B., Richardson J.P., Naglik J.R. (2020). Candidalysin is a potent trigger of alarmin and antimicrobial peptide release in epithelial cells. Cells.

[32] Sham D., Wesley U.V., Hristova M., Van Der Vliet A. (2013). ATP-mediated transactivation of the epidermal growth factor receptor in airway epithelial cells involves DUOX1-dependent oxidation of Src and ADAM17. PLoS One.

[33] Swidergall M., Solis N.V., Lionakis M.S., Filler S.G. (2018). EphA2 is an epithelial cell pattern recognition receptor for fungal β-glucans. Nat. Microbiol..

[34] Nikou S.A., Zhou C., Griffiths J.S., Kotowicz N.K., Coleman B.M., Green M.J. (2022). The Candida albicans toxin candidalysin mediates distinct epithelial inflammatory responses through p38 and EGFR-ERK pathways. Sci. Signal..

[35] Westman J., Plumb J., Licht A., Yang M., Allert S., Naglik J.R. (2022). Calcium-dependent ESCRT recruitment and lysosome exocytosis maintain epithelial integrity during Candida albicans invasion. Cell Rep..

[36] Blagojevic M., Camilli G., Maxson M., Hube B., Moyes D.L., Richardson J.P. (2021). Candidalysin triggers epithelial cellular stresses that induce necrotic death. Cell. Microbiol..

[37] Swidergall M., Filler S.G. (2017). Oropharyngeal candidiasis: fungal invasion and epithelial cell responses. PLoS Pathog..

[38] Naglik J.R., König A., Hube B., Gaffen S.L. (2017). Candida albicans–epithelial interactions and induction of mucosal innate immunity. Curr. Opin. Microbiol..

[39] Verma A.H., Richardson J.P., Zhou C., Coleman B.M., Moyes D.L., Ho J. (2017). Oral epithelial cells orchestrate innate type 17 responses to Candida albicans through the virulence factor candidalysin. Sci. Immunol..

[40] Conti H.R., Peterson A.C., Brane L., Huppler A.R., Hernández-Santos N., Whibley N. (2014). Oral-resident natural Th17 cells and γδ T cells control opportunistic Candida albicans infections. J. Exp. Med..

[41] Conti H.R., Shen F., Nayyar N., Stocum E., Sun J.N., Lindemann M.J. (2009). Th17 cells and IL-17 receptor signaling are essential for mucosal host defense against oral candidiasis. J. Exp. Med..

[42] Conti H.R., Bruno V.M., Childs E.E., Daugherty S., Hunter J.P., Mengesha B.G. (2016). IL-17 receptor signaling in oral epithelial cells is critical for protection against oropharyngeal candidiasis. Cell Host Microbe.

[43] Aggor F.E.Y., Break T.J., Trevejo-Nuñez G., Whibley N., Coleman B.M., Bailey R.D. (2020). Oral epithelial IL-22/STAT3 signaling licenses IL-17-mediated immunity to oral mucosal candidiasis. Sci. Immunol..

[44] Obel J.D. (1997). Vaginitis. N. Engl. J. Med..

[45] Willems H.M.E., Ahmed S.S., Liu J., Xu Z., Peters B.M. (2020). Vulvovaginal candidiasis: a current understanding and burning questions. J. Fungi (Basel).

[46] Yano J., Kolls J.K., Happel K.I., Wormley F., Wozniak K.L., Fidel P.L. (2012). The acute neutrophil response mediated by S100 alarmins during vaginal Candida infections is independent of the Th17-pathway. PLoS One.

[47] Peters B.M., Coleman B.M., Willems H.M.E., Barker K.S., Aggor F.E.Y., Cipolla E. (2020). The interleukin (IL) 17R/IL-22R signaling axis is dispensable for vulvovaginal candidiasis regardless of estrogen status. J. Infect. Dis..

[48] Bruno V.M., Shetty A.C., Yano J., Fidel P.L., Noverr M.C., Peters B.M. (2015). Transcriptomic analysis of vulvovaginal candidiasis identifies a role for the NLRP3 inflammasome. mBio.

[49] Jaeger M., Carvalho A., Cunha C., Plantinga T.S., van de Veerdonk F., Puccetti M. (2016). Association of a variable number tandem repeat in the NLRP3 gene in women with susceptibility to RVVC. Eur. J. Clin. Microbiol. Infect. Dis..

[50] Borghi M., de Luca A., Puccetti M., Jaeger M., Mencacci A., Oikonomou V. (2015). Pathogenic NLRP3 inflammasome activity during Candida infection is negatively regulated by IL-22 via activation of NLRC4 and IL-1Ra. Cell Host Microbe.

[51] Leemans J.C., Cassel S.L., Sutterwala F.S. (2011). Sensing damage by the NLRP3 inflammasome. Immunol. Rev..

[52] Lowes D.J., Hevener K.E., Peters B.M. (2020). Second-generation antidiabetic sulfonylureas inhibit Candida albicans and Candidalysin-mediated activation of the NLRP3 inflammasome. Antimicrob. Agents Chemother..

[53] Rogiers O., Frising U.C., Kucharíková S., Jabra-Rizk M.A., van Loo G., van Dijck P. (2019). Candidalysin crucially contributes to nlrp3 inflammasome activation by Candida albicans hyphae. mBio.

[54] Kasper L., König A., Koenig P.A., Gresnigt M.S., Westman J., Drummond R.A. (2018). The fungal peptide toxin Candidalysin activates the NLRP3 inflammasome and causes cytolysis in mononuclear phagocytes. Nat. Commun..

[55] Roselletti E., Perito S., Gabrielli E., Mencacci A., Pericolini E., Sabbatini S. (2017). NLRP3 inflammasome is a key player in human vulvovaginal disease caused by Candida albicans. Sci. Rep..

[56] Alonso-Monge R., Gresnigt M.S., Román E., Hube B., Pla J.s. (2021). Candida albicans colonization of the gastrointestinal tract: a double-edged sword. PLoS Pathog..

[57] de Luca A., Montagnoli C., Zelante T., Bonifazi P., Bozza S., Moretti S. (2007). Functional yet balanced reactivity to Candida albicans requires TRIF, MyD88, and IDO-dependent inhibition of Rorc. J. Immunol..

[58] Kumamoto C.A. (2011). Inflammation and gastrointestinal Candida colonization. Curr. Opin. Microbiol..

[59] Pande K., Chen C., Noble S.M. (2013). Passage through the mammalian gut triggers a phenotypic switch that promotes Candida albicans commensalism. Nat. Genet..

[60] Allert S., Förster T.M., Svensson C.-M., Richardson J.P., Pawlik T., Hebecker B. (2018). Candida albicans-induced epithelial damage mediates translocation through intestinal barriers. mBio.

[61] Zhai B., Ola M., Rolling T., Tosini N.L., Joshowitz S., Littmann E.R. (2020). High-resolution mycobiota analysis reveals dynamic intestinal translocation preceding invasive candidiasis. Nat. Med..

[62] Marco F., Lockhart S.R., Pfaller M.A., Pujol C., Rangel-Frausto M.S., Wiblin T. (1999). Elucidating the origins of nosocomial infections with Candida albicans by DNA fingerprinting with the complex probe Ca3. J Clin. Microbiol..

[63] Li X.v., Leonardi I., Putzel G.G., Semon A., Fiers W.D., Kusakabe T. (2022). Immune regulation by fungal strain diversity in inflammatory bowel disease. Nature.

[64] Lazaridis L.D., Pistiki A., Giamarellos-Bourboulis E.J., Georgitsi M., Damoraki G., Polymeros D. (2017). Activation of NLRP3 inflammasome in inflammatory bowel disease: differences between Crohn’s disease and ulcerative colitis. Dig. Dis. Sci..

[65] van Thiel I.A.M., Stavrou A.A., de Jong A., Theelen B., Davids M., Hakvoort T.B.M. (2022). Genetic and phenotypic diversity of fecal Candida albicans strains in irritable bowel syndrome. Sci. Rep..

[66] Pappas P.G., Lionakis M.S., Arendrup M.C., Ostrosky-Zeichner L., Kullberg B.J. (2018). Invasive candidiasis. Nat. Rev. Dis. Primers.

[67] Bechinger B. (1997). Structure and functions of channel-forming peptides: magainins, cecropins, melittin and alamethicin. J. Membr. Biol..

[68] Shai Y. (2002). Mode of action of membrane active antimicrobial peptides. Biopolymers.

[69] Avci F.G., Akbulut B.S., Ozkirimli E. (2018). Membrane active peptides and their biophysical characterization. Biomolecules.

[70] Bischofberger M., Iacovache I., Gisou Van Der Goot F. (2012). Pathogenic pore-forming proteins: function and host response. Cell Host Microbe.

[71] Peraro M.D., van der Goot F.G. (2016). Pore-forming toxins: ancient, but never really out of fashion. Nat. Rev. Microbiol..

[72] Scott H.L., Nguyen V.P., Alves D.S., Davis F.L., Booth K.R., Bryner J. (2015). The negative charge of the membrane has opposite effects on the membrane entry and exit of pH-low insertion peptide. Biochemistry.

[73] Tuerkova A., Kabelka I., Králová T., Sukeník L., Pokorná Š., Hof M. (2020). Effect of helical kink in antimicrobial peptides on membrane pore formation. Elife.

[74] Strehlow K.G., Baldwin R.L. (1989). Effect of the substitution Ala .fwdarw. Gly at each of five residue positions in the C-peptide helix. Biochemistry.

[75] Lifson S., Roig A. (1961). On the theory of helix-coil transition in polypeptides. J. Chem. Phys..

[76] Strehlow K.G. (2006). Effect of alanine versus glycine in alpha-helices on protein stability. Proteins.

[77] Abderemane-Ali F., Rossen N.D., Kobiela M.E., Craig R.A., Garrison C.E., Chen Z. (2021). Evidence that toxin resistance in poison birds and frogs is not rooted in sodium channel mutations and may rely on “toxin sponge” proteins. J. Gen. Physiol..

